# A Systematic Review of Cognitive and Behavioural Symptoms in CTNNB1 Syndrome

**DOI:** 10.1007/s11065-025-09660-y

**Published:** 2025-02-20

**Authors:** Mercè Pallarès-Sastre, Imanol Amayra, Monika Salgueiro, Elena Villanueva-Viar, Amaia Lasa-Aranzasti, Maitane García

**Affiliations:** 1 Neuro-E-Motion Research Team, Department of Psychology, Faculty of Health Sciences, University of Deusto, Avenida de Las Universidades 24, 48007 Bilbao, Spain; 2 Department of Clinical and Health Psychology and Research Methodology, Faculty of Psychology, University of the Basque Country UPV/EHU, Donostia, Spain; 3 Department of Clinical and Molecular Genetics, Vall d’Hebron University Hospital, Barcelona, Spain

**Keywords:** CTNNB1 syndrome, Features of autism, Intellectual disability, Language impairments, Neurodevelopmental disorder

## Abstract

CTNNB1 syndrome is a rare neurodevelopmental disorder caused by a likely pathogenic or pathogenic variant in the *CTNNB1* gene. A systematic review was conducted to examine previous research that provided CTNNB1 syndrome patients, specifically those that described intellectual quotient, motor development, language impairments, behavioural problems and features of autism. Databases examined were PubMed and Scopus. The inclusion criteria were (a) reported human patients diagnosed with CTNNB1 syndrome by a genetic test; (b) were related to cognition, intelligence quotient, motor development, language impairment, behavioural problems or features of autism; (c) did not have another genetic diagnosis and (d) were written in Spanish or English. A total of 42 studies were included. Overall, the symptomatology described was very heterogeneous with varying degrees of impairment among patients. However, individuals reached most significant developmental milestones later than expected and with different degrees of impairment. The use of standardised methodology to assess cognitive and behavioural domains was scarce in most studies, and the vast majority did not include a specific assessment protocol based on the symptomatology of CTNNB1 syndrome individuals. In addition, only two adult patients were described in depth, which implies that there are many unknowns about the progression of the syndrome later in life. Therefore, future research should focus on increasing the sample assessed and count with a standardised protocol in order to characterise the cognitive and behavioural phenotype of CTNNB1 syndrome.

CTNNB1 syndrome is a severe neurodevelopmental disorder caused by a de novo genetic variant (Tucci et al., 2014). A great phenotypic variety has been detected in the patients reported so far, yet the main features described by researchers are global cognitive impairment, abnormal muscle tone, delayed acquisition of language and motor skills, behaviour problems and autistic spectrum disorder (ASD) symptoms, morphological alteration of the eye, microcephaly and mild dysmorphic features (Dubruc et al., 2014; Kharbanda et al., 2017; Kuechler et al., 2015). De Ligt et al. (2012) were the first to implicate the *CTNNB1* gene in autosomal dominant intellectual disability (ID) using exome sequencing. Subsequently, several studies have been published in the literature that have provided more knowledge about the syndrome, leading to a preliminary first phenotypic characterisation of the disease (Kuechler et al., 2015).

From a genetic perspective, the *CTNNB1* gene is located on chromosome 3 and is composed of 16 exons. Exons 2 to 15 are responsible for encoding the β-catenin protein. This protein consists of 781 amino acids and is part of the armadillo protein family, which has a structural role in embryonic development (Li et al., 2017). It belongs to the cadherin complex, which is involved in cell–cell adhesion, and is also part of the Wnt signalling pathway. In particular, the Wnt/β-catenin signalling is known to be essential for the development and functional of the mammalian central nervous system (Caracci et al., 2021). Both overexpression and decrease of β-catenin affect the neuronal synapse regulation and remodelling of the nervous system, having a direct impact on learning and explicit memory processes (Maguschak and Ressler 2012). Given its relatively recent discovery and the low prevalence of CTNNB1 syndrome, little is known about the effect of the variant and the relationship between gene expression and disease severity. The strongest hypothesis is that the variant results in partial loss of protein function, although another possible hypothesis points to a complete loss of function, either through non-coding or translation of non-functional proteins (Miroševič et al., 2022).

Current treatments available for neurodevelopmental disorders, and specifically for CTNNB1 syndrome, are mainly palliative and focus on cognitive stimulation and improvement of behavioural symptoms and motor impairments. However, the lack of research prevents the detection of specific needs of this disease, and consequently, there are no intervention programmes designed to enhance the preserved cognitive domains and stimulate the impaired ones.

This syndrome presents heterogeneous phenotypes, usually diagnosed during the early years and persisting throughout life (Wilfert et al., 2017). As mentioned before, individuals affected by the disease present a wide variety of symptomatology, which makes it difficult to establish common features among patients. Nevertheless, Miroševič et al. (2022) carried out a first attempt to correlate genotype and phenotype including the case series published in the literature, making a classification according to four domains: eye contact, language, cognition and motor development. They proposed six degrees of severity, from normal to severe symptomatology. Although it is a preliminary study, the sample was too small to obtain significant correlations and the evaluation criteria were not consistent between studies. It is considered the first study to describe the genotype–phenotype relationship of CTNNB1 syndrome and the starting point for future research (Miroševič et al., 2022).

Another of the most relevant studies in the current literature was carried out by Kayumi et al. (2022), who studied a cohort of 120 individuals diagnosed with CTNNB1 syndrome and established that some of the most common neurological traits were ID (94.1%), motor delay (93.7%), delayed speech and language development (90.4%) and behavioural abnormalities (74.2%). However, the authors did not report the use of standardised methods to obtain such conclusions in the majority of variables. Nevertheless, these results show the high prevalence of these symptoms and, consequently, claim the need to study them in depth. Therefore, the purpose of this study was to perform a comprehensive literature review to examine the cumulative evidence of cognitive and behavioural impairments in CTNNB1 syndrome, specifically of those studies that have used standardised or systematic methods to do so, and draw a cognitive and behavioural profile of the disease from the data reported so far.

## Methods

This systematic review was conducted according to the guidelines of “Preferred Information Elements for Systematic Reviews and Meta-Analysis” (PRISMA).

### Data Sources and Search Strategy

Databases included were PubMed and Scopus, whereas the searching terms were (1) CTNNB1, (2) CTNNB1 syndrome, (3) *CTNNB1* gene, (4) CTNNB1 mutation, (5) Cognitive, (6) Intellectual disability, (7) neurodevelopmental disorder and (8) beta-catenin. The combinations made for the search strategy are reflected in Table 1 and were exclusive within the article title, the abstract and the keywords.

**Table 1:** Search strategy for the databases

Set	Search string
1	CTNNB1 AND Cognitive
2	CTNNB1 AND “Neurodevelopmental disorder”
3	CTNNB1 AND “Intellectual disability”
4	“CTNNB1 syndrome” AND Cognitive
5	“CTNNB1 gene” AND “Intellectual disability”
6	“CTNNB1 mutation” AND “Intellectual disability”
7	“CTNNB1 mutation” AND “Neurodevelopmental disorder”
8	Beta-catenin AND “Intellectual disability”

### Inclusion and Exclusion Criteria

Studies were included from inception to the 6th of March 2024. We included articles that (a) reported human patients diagnosed with *CTNNB1* pathogenic or likely pathogenic variance only according to the American College of Medical Genetics guideline and excluded variant of uncertain significance; (b) were related to cognition, intelligence quotient (IQ), language impairment, motor development, features of autism or behavioural problems and (c) did not have another genetic disorder causing neurodevelopmental symptoms. Literature reviews, meta-analysis, conference papers and studies on cell and animal models were excluded.

### Identification of Relevant Studies and Data Extraction

**Fig. 1:**
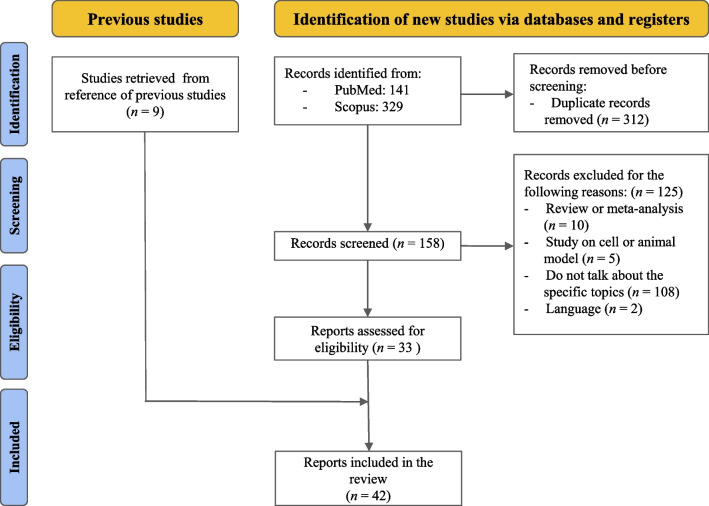
Flow diagram of the literature search

The search strategy identified 470 articles, and four studies were included by hand-search (Coussa et al., 2020; Dixon et al., 2016; Sun et al., 2019; Taylor et al., 2022). After removing duplicates and those that did not meet inclusion criteria, there were 42 eligible articles left (see Fig. 1 for flow diagram). Studies that met inclusion criteria were summarised according to (i) the authors, (ii) sample used, (iii) chosen clinical features, (iv) instruments used for evaluation and (v) a summary of the results obtained (Table 2).

**Table 2:** Literature review of cases with CTNNB1 syndrome

Author	Sample	Variables	Instrument	Results
Dubruc et al. (2014)	Case report • Age: 5 ½ • Gender: F	IQ	-	Intellectual disability • 4 yo and 6 mo: counted to 10 and understood simple instructions
		Motor	-	• 14 mo: sat unaided • 30 mo: was able to stand • 3 yo and 2 mo: had truncal hypotonia • 3 yo and 7 mo: walked with aid and normal fine motor skills • 4 yo and 6 mo and at 5 yo and 6 mo: was able to take a few steps unaided. Presented ataxic gait and spasticity of the lower limbs with brisk, polykinetic deep tendon reflexes and a Babinski response
		Language	-	• 3 yo and 2 mo: was able to say 50 words and combine 2 words. Better comprehension than expression skills. Orofacial dyspraxia • 4 yo and 6 mo: able to combine several words
		Behavioural traits	-	• 3 yo and 2 mo: although friendly and sociable, presented hyperactivity symptoms and focus difficulties for long periods
Tucci et al. (2014)	*N* = 4 (2 of them previously reported by de Ligt et al. (2012)) • Case 1: 4 yo 6 mo/M • Case 2: 29 yo/F • Case 3: 51 yo/F • Case 4: 14 yo/F	IQ	-	Case 2: • 6 mo: since then very slow cognitive development • 29 yo: moderate to severe ID Case 3: • 50 yo: cognitive abilities gradually deteriorated Case 4: • 7 yo: an intelligence test gave an IQ score of 65 • 14 yo: intellectual functioning corresponded to a level of mild to moderate
		Motor	-	Case 1: • Neonatal: delayed psychomotor development and low muscle tone • 18 mo: sat independently • 4 yo and 6 mo: walk short distances but preferred to move on his knees Case 2: • 6 mo: developmental regression and hypotonia • 2 yo: sat without support • 3 yo: started to crawl • 12 yo: was able to walk with little support and presented progressive spasticity Case 3: • Delayed motor development and able to walk with a walking frame • 50 yo: presented progressive spasticity and was no longer able to walk • 51 yo: severely disabled and fully wheelchair dependent Case 4: • Neonatal: hypotonia and developmental delay (6 mo) • 12 mo: sat without support • 13 mo: diagnosed with spastic diplegia of the lower extremities • 4 yo and 5 mo: walked with aid and used a wheelchair for longer distances
		Language	-	Case 1: • 30 mo: communicated with pictograms • 4 yo and 6 mo: spoke some sentences, articulated poorly and was hard to understand Case 2: • 9/10 yo: spoke her first words • 29 yo: able to speak a few single words Case 3: • Not able to speak but used sign language Case 4: • 3 yo: started to babble • 14 yo: spoke in simple sentences and read simple words
		Behavioural traits	-	Case 2: • Aggression, auto-mutilation and faecal smearing
		Features of autism	-	Case 4: • Showed autism spectrum disorder features
Kuechler et al. (2015)	*N* = 16 (novel patients from 15 families) • Age range: 1$\frac{3}{12}$–15$\frac{4}{12}$ • Gender: F, 8; M, 8	IQ	-	• Intellectual disability: 15 out of 16. One case was considered moderate and another was too young
		Motor	-	• Motor delay: severe (9/15); moderate (4/15); mild (3/15) • Truncal hypotonia: 14 out of 16. One refers truncal ataxia • Peripheral hypertonia/spasticity: 15 out of 16. Other refer ataxic gait, dystonia and further affectation on the legs than the arms • Crawling (mo): 14, 23, 24, 17, 12, 18, 25 • Free walking ability: 6 out of 15 • In need of aid to walk: 4 out of 16
		Language	-	• No words: 5 out of 16 (1 was still young) • Severe (few words): 5 out of 16 • Moderate: 3 out of 16 • Mild (full sentences but delayed speech): 3 out of 16 • Basic speech comprehension: 15 out of 16 (1 bilingual and the other case still too young)
		Behavioural traits	-	• Good social interaction: 9 out of 16 • Temper tantrums: 4 out of 16 • Sleep disturbances: 3 out of 16 • Aggressions/auto-aggressions: 6 out of 16 • ADHD: 2 out of 16
		Features of autism	-	• Features of autism: 3 out of 16 • Little eye contact: 2 out of 16 • Stereotypic movements: 3 out of 16
Dixon et al. (2016)	Case report • Age: 22 mo • Gender: M	IQ	-	Intellectual disability
		Motor	-	Developmental delay
		Features of autism	-	Behaviours on the autism spectrum
Prasad et al. (2016)	*N* = 1 • Age: 3 yo • Gender: F	IQ	-	Intellectual disability
Retterer et al. (2016)	*N* = 5	IQ	-	Intellectual disability
Thevenon et al. (2016)	*N* = 1 • Age: 14 yo • Gender: M	IQ	-	Severe intellectual disability
		Motor	-	Spastic paraplegia and dystonia
		Features of autism	-	Features of autism
Winczewska-Wiktor et al. (2016)	Case report • Age: 11 yo • Gender: M	IQ	-	Mild intellectual disability
		Motor	-	• 20 mo: delayed psychomotor development and unable to walk unassisted. Sudden loss in muscle tone of lower limbs that lead to episodic falls, diagnosed with hyperekplexia. Left hemiparesis with positive Babinski sign and mild dysarthria • 3 yo: free walking but did not previously walk • 11 yo: coordination problems and needs support to walk. Truncal hypotonia and peripheral hypertonia/spasticity
		Language	-	• 20 mo: delayed speech development, only able to speak a few words • 11 yo: increased problems to speak and unclear speech. Basic speech comprehension preserved
		Behavioural traits	-	• 20 mo: normal social and emotional developmental, but hyperactive • 11 yo: friendly, anxious, verbal aggressions, temper tantrums, crying and sleep disturbances
		Features of autism	-	Avoided eye contact, stereotypic movements and had episodes of frustration
Kharbanda et al. (2017)	*N* = 10 • Age range: 3–27 • Gender: F, 7; M, 3	Motor	-	All of the patients presented significant motor delay. 7 out of 10 patients started walking from 30 mo to 4–5 yo, but with ataxic symptoms. The remaining sample could not walk at 14, 6$\frac{2}{12}$and 3$\frac{3}{12}$
		Language	-	All of the patients have significant speech delay but with a lot of variability • No words: 2 out of 10 • Few words and delayed acquisition (around 4 or 5 yo with improvement of speech at 6 yo): 6 out of 10 • Spoke in sentences: 2 out of 10 (1 case was 11 yo and the other 1 an adult, whose speech was unclear)
		Behavioural traits	-	• Temper tantrums: 4 out of 10 • Sleep disturbances: 2 out of 10 • Aggressions/auto-aggressions: 5 out of 10 • ADHD: 1 out of 10
		Features of autism	-	• Features of autism: 1 out of 10 • Stereotypic movements: 3 out of 10
Krupp et al. (2017)	*N* = 1 • Gender: M	IQ	-	• Nonverbal IQ: 106 • Verbal IQ: 86
		Features of autism	-	Features of autism
Li et al. (2017)	Case report • Age: 15 mo • Gender: M	IQ	-	Too young to determinate
		Motor	-	• 9 mo: could not roll or sit (motor delay). Presented mild thumb adduction and hypertonia of the extremities • 15 mo: able to raise his head but still could not sit or walk without support
		Language	-	• 15 mo: no language development although he was still too young
		Behavioural traits	-	Too young to determinate
Panagiotou et al. (2017)	Case report • Age: 3 yo • Gender: M	Motor	-	Motor delay
		Language	-	Significant speech delay (use of 1 or 2 single words and most communication was nonverbal)
Cordeiro et al. (2018)	*N* = 1 • Age: 8 yo • Gender: M	IQ	-	Global developmental delay
		Motor	-	Dystonia. After treatment (L-dopa/carbidopa (5.2 kg/d)) improved dystonia
Percy et al. (2018)	Case report • Age: 15 yo • Gender: F	IQ	-	Cognitive delay
		Motor	-	• 6 mo: floppy • 3 yo: suffered a motor regression; lost pincer grasp and finger feeding • 15 yo: walked with a broad and had dyspraxic gait. Diagnosed with appendicular hypotonia and moderate bradykinesia, but normal strength and muscle stretch reflexes
		Language	-	• 3 yo: worsening of expressive and receptive abilities • 15 yo: not vocal
		Behaviour traits	-	• 6 mo: frequently irritable • 5 yo: self-aggression (biting)
		Features of autism	-	• 1 yo: presented hand-flapping and hand-clapping • 15 yo: alert and interactive but not vocal, gave eye contact 25% of the time
Pipo-Deveza et al. (2018)	Case report • Age: 4 y 11 mo • Gender: F Effects of L-dopa/carbidopa (100/25) (mg/kg/day of L-dopa)	IQ	-	• (L-Dopa second does) 3 yo and 10 mo: recognised the alphabet • (L-Dopa final doses) 4 yo and 11 mo: recognised letters and single words, counted to 60 and acquisition of cognitive to age-appropriate skills
		Motor	Tardieu scale^b^; GMFM 88^b^; 6MWT^b^	Delayed gross motor skills: • 15/16 mo: sat unsupported • 19 mo: stood • 18 mo: had a pincer and attempted buttons and zippers • 2 yo: began tip toe gait with aid of walker and orthotics • (L-Dopa baseline) 3 yo and 5 mo: axial hypotonia, marked hypertonia involving legs > arms with dystonia, spasticity, hyperlexia, intoeing and upgoing plantars. She had tiptoe gait and was walker-dependant and required AFOs • (L-Dopa second doses) 3 yo and 10 mo: walked 30 steps without support and improved dystonia • (L-Dopa final doses) 4 yo and 11 mo: significant reduction of hypertonia on the Tardieu scale and increased mobility on the GMFM 88 and the 6MWT score. Acceleration in acquisition of fine motor skills
		Language	-	• 14 mo: first word • 17 mo: stopped speaking • (L-Dopa baseline) 3 yo 5 mo: regained speech thanks to speech therapy (300 words) and spoke in 9- to 10-word sentences with dysarthria • (L-Dopa second doses) 3 yo 10 mo: > 500 words and spoke in 10-word sentences • (L-Dopa final doses) 4 yo 11 mo: recited the alphabet and spelt her name and acquisition of language to age-appropriate skills
		Behaviour traits	-	• 3 yo and 5 mo: stranger anxiety
Takezawa et al. (2018)	*N* = 1 • Age: 7 yo • Gender: M	IQ	-	Intellectual disability
		Motor	GMFCS; MACS	• GMFCS: 4 • MACS: 3 • Spastic diplegia and hyperekplexia
		Language	CFCS	• CFCS: 3
		Features of autism	-	No features of autism
Sun et al. (2019)	*N* = 4 • Case 1: 15 mo/F • Case 2: 4 yo/F • Case 3: 4 yo/M • Case 4: N/A/M	IQ	-	Case 1, 2 and 3: learning disabilities
		Motor	-	Case 1, 2 and 3: motor delay and scoliosis. Case 1 had difficulties controlling her head
		Language	-	Case 1: unable to speak at 15 mo
		Features of autism	-	Case 1, 2 and 3: exhibit autism
Wang et al. (2019)	*N* = 2 (mother and daughter) • Case 1: 27 yo • Case 2: 49 yo	IQ	WAIS^b^	• Case 1: test showed severe ID with an IQ score of 28 • Case 2: test showed severe ID with and IQ score of 40
		Motor	-	Significant motor delay. Case 1 had peripheral hypertonia with deep tendon hyperreflexia and Babinski sign positive, ataxic gait and paroxysmal dystonic of head and neck
		Language	-	Both show language retardation
Coussa et al. (2020)	Case report • Age: 8 mo • Gender: N/A	Motor	-	• 8 mo: mild motor delay
Jin et al. (2020)	*N* = 3 • Case 1: 11 yo/F • Case 2: M • Case 3: F	IQ	-	Intellectual disability
		Motor	GMFCS	All patients presented spasticity • Case 1: spasticity concentrated in her lower limbs, dystonia, gait abnormality and used AFOs for mobility. GMFCS level of 2 • Case 2: spasticity and GMFCS level of 3
		Language	-	Language disorders
		Behaviour traits	-	Behaviour problems • Case 1: explosive behaviour and ADHD
Ke and Chen (2020)	Case report • Age: 15 mo • Gender: F	IQ	-	Lower intelligence compared to her peers
		Motor	-	• 3 mo: could not raise her head (delayed motor development) • 15 mo: could raise her head (thanks to 4 mo of rehabilitation training) but unstably and could not sit. Significant decrease of the tension of the trunk and peripheral limbs, also with weakened bilateral tendon reflexes. Presented hypotonia
		Language	-	• 15 mo: made some sounds but could not speak. After 4 mo of rehabilitation training, there were improvements in the capacity to speak
Rossetti et al. (2020)	*N* = 7 • Case 1: 23 mo/M • Case 2: 7 yo/M • Case 3: 11 yo/F • Case 4: 3 yo/F • Case 5: 4 yo/F • Case 6: 5 yo/M • Case 7: 8 yo/M	Motor	-	All were globally developmentally delayed • Case 1: was hypotonic in the trunk and upper extremities but spastic in the lower extremities • Case 2: diagnosed with restless leg syndrome. On exam was noted to have bradykinesia and toe walking, as well as hypertonia in the lower extremities • Case 3: physical exam showed overall hypotonia but had spasticity in the lower extremities bilaterally • Case 4: on exam, was hypotonic, although her tone seemed to improve over time • Case 5: on exam, he showed truncal hypotonia with hypertonicity in the distal extremities. He also presented choreiform movements and intention tremor • Case 6: physical exam showed hypotonia with spasticity in the lower extremities • Case 7: on exam, was hypotonic
		Language	-	• Case 3: articulation issues but otherwise normal speech development • Case 6 and 7: absent speech
		Behaviour traits	-	• Case 2: bit and pinched other people • Case 5: self-injurious behaviours • Case 7: screaming spells and self-injurious behaviours
		Features of autism	-	• Case 2 and 3: repetitive behaviours (specifically for case 3; hand-wringing and nail-picking) • Case 5: diagnosed with autism spectrum disorder
Verhoeven et al. (2020)	Case report • Age: 32 yo • Gender: F	IQ	VABS^a^	General developmental delay • 7 mo: special education and individual guidance within a structured environment were recommended • VABS: at the daily activities domain she obtained a punctuation in accordance with 44 mo
		Motor	VABS^a^	• 7 mo: physical therapy was advised to stimulate motor development • 11 mo: head and truncal instability • 17 mo: suboptimal head balance, absent truncal balance and inability to sit or walk. Prescription of a Pavlik harness for hip dysplasia. Physical therapy led to gradual but limited improvement of motor skills • 7 yo: marked hypotonia with an uncoordinated global movement pattern • 9 yo: was able to walk with support for a short distance, although she usually used a wheeled walker and was partially wheelchair bound • 14 yo: she developed a spastic ataxic walking pattern • 21 and 32 yo: progressive problems with walking (completely wheelchair bound) and was only able to take a few steps with a broad-based gait • VABS: at the motor skill domain, she obtained a punctuation in accordance with 16 mo
		Language	VABS^a^	• 7 mo: general refusal of communication • 17 mo: logopedic therapy led to gradual but limited improvement of speech and language • 7 yo: speech was restricted to 2–4 words and receptive language was about 3–4 yo • VABS: at the communication domain, she obtained a punctuation in accordance with 34 mo
		Behaviour traits	-	• 7 yo: challenging and ritualistic behaviours with temper tantrums and mood instability. Communication was limited by frequent crying, involuntary gestures and head banging • 21 yo: behaviours with temper tantrums, anger and screaming especially in situations with less structure and predictability • Outcome and follow-up: recommendation of daily guidance that resulted in a significant reduction of her challenging behaviours (aggression, temper tantrums and anxieties)
		Features of autism	VABS^a^; SED-R^a^; AVZ-R^a^; ADOS-2^a^	• 7 mo: lack of eye contact • 17 mo: autistic behaviours were noted (confirmation at 7 yo and diagnosed at 12 yo) • VABS: at the socialisation domain, she obtained a punctuation in accordance with 18 mo • SEO-R: developmental age of 18 mo • AVZ-R: score of 16, autisms signs detected • ADOS-2: score of 27 corroborated an autism diagnosis
Yan et al. (2020)	Case report • Age: 18 mo • Gender: M	IQ	-	Intellectual disability
		Motor	-	Peripheral hypertonia and delayed motor development
		Language	-	Delayed speech
Bulot et al. (2022)	*N* = 12 • Age range: 2–19 One as a case report • Age: 19 yo • Gender: F	IQ	Online questionnaire	• Intellectual disability: 8 out of 12 (the others did not refer) • Severe learning difficulties: 4 out of 8. Diversity in the ability to read or talk • Learning difficulties: 4 out of 8. Most were able to read, talk and do some simple maths
		Motor	Online questionnaire	All mentioned motor delay and gait disorders • Free siting (11 cases): mean age at 17 mo (ranged from 8 to 30 mo) • Free walking (11 cases): age ranged from 24 mo to 14 yo
		Language	Online questionnaire	All cases were described with speech delay. First words were acquired at 2 yo approximately but speech progression varied among cases. The majority reported good comprehension, even some cases were able to understand complex orders. Some cases mentioned articulation difficulties
		Behaviour traits	Online questionnaire	• Good social interaction: 4 out of 12 (more difficulties with other kids than adults) • Temper tantrums: 9 out of 12 • Aggressions/auto-aggressions: 6 out of 12 • Quiet child: 4 out of 12 • ADHD: 2 out of 12
		Features of autism	Online questionnaire	• Autistic diagnosed: 2 out of 12 • Little eye contact: 4 out of 12 • Repetitive behaviours: 8 out of 12 • Interest in specific things: 6 out of 12
Dashti et al. (2022)	Case report • Age: 8 yo • Gender: F	IQ	WISC-V^b^	Non-progressive low average score (80–89)
		Motor	-	• 9 mo: hypotonia and significant motor delay • 8 yo: truncal hypotonia, spasticity and dystonic movements of the lower extremities. Support to walk. Most affected part of the body with weakness and stiffness was the lower limb on both sides. In need of occupational therapy
		Language	-	• 5 yo: first word achieved (speech delay). Speech therapy (10 sessions) helped considerably on improving speech ability
		Features of autism	-	Adequate social interaction and eye contact, sensitive to loud noises, obsessive behaviour, tics and agitation
Ho et al., (2022a, 2022b)	*N* = 9 • Age range: 3–37 • Mean age: 13.44 yo • Gender: F, 4; M, 5 (One case by Panagiotou et al. (2016))	IQ	-	All patients had developmental delay and special education needs. There was no developmental regression in the cohort • IQ assessments (did not mention which ones) were administered to 2 cases, showing moderate intellectual disability • Mild intellectual disability: 1 out of 9 • Moderate intellectual disability: 6 out of 9 Moderate-severe intellectual disability: 1 out of 9
		Motor	-	• Hypotonia: 6 out of 9 (before being 1 yo) • Progressive peripheral spasticity: 8 out of 9 • Brisk jerks: 8 out of 9 • Dystonia: 4 out of 9 (age of onset from 17 mo to 4 yo). One case had variable dystonia that affected only the upper or lower limbs
Kayumi et al. (2022)	*N* = 56 • Gender: F, 35; M, 29 (Combined with 68 previously published)	IQ	-	Intellectual disability/developmental delay (111/118, 89.8–98.3%)
		Motor	-	• Motor delay (104/111, 89.2–98.2%) • Truncal hypotonia (80/93, 79– 93.1%) • Peripheral spasticity or hypertonia (81/104, 69.9–85.9%)
		Language	-	Delayed speech and language development (104/115, 85.1–95.8%)
		Behaviour traits	-	Behavioural abnormalities (69/93, 65–83.1%)
Lee et al. (2022)	*N* = 13 • Case 1: 5 yo/M • Case 2: 20.5 yo/F • Case 3: 7 yo/M • Case 4: 11 yo/M • Case 5: 8.5 yo/F • Case 6: 10 yo/M • Case 7: 9.5 yo/M • Case 8: 10 yo/F • Case 9: 8 yo/M • Case 10: 7.5 yo/M • Case 11: 6 yo/F • Case 12: 8.5 yo/F • Case 13: 4 yo/M	IQ	-	Variant degrees of intellectual disability among 3 patients, with results ranging from 40 (case 2) to 58 (case 12)
		Motor	-	• Sits with support: 2 out of 13 • Walk with support: 3 out of 13 • Walk alone: 8 out of 13 • Spastic gait: 3 out of 13 • Ataxic gait: 4 out of 13 • Dyspraxic gait: 1 out of 13 • Tiptoe gait: 6 out of 13
		Language	-	• No words: 2 out of 13 • No words except mama and papa: 2 out of 13 • < 10 single words: 3 out of 13 • Sentences: 5 out of 13 • Able to read: 5 out of 13 • Dysarthria: 2 out of 13
		Behaviour traits	-	Behavioural problems described in 6 out of 13 participants • Aggressive behaviour: 4 out of 13 • Attention deficit: 1 out of 13 • Hyperactivity: 2 out of 13 • Impulsivity: 2 out of 13
		Features of autism	-	Features of autism mentioned in 3 out of 13 participants • Hand stereotypy: 2 out of 13 • Bruxism: 2 out of 13 • Breathing irregularity: 1 out of 13
Paparella et al. (2022)	Case report • Age: 13 • Gender: F	IQ	-	• First month: psychomotor delay • 6 yo: mild intellectual disability • 10 yo: intellectual disability worsened (TIQ < 40)
		Motor	-	• First month: delayed motor milestones and hypotonia • 6 yo: difficulties in motor coordination • 8 yo: ataxic gait
		Language	-	Language delay • 6 yo: speech delay in both expression and comprehension
		Behaviour traits	-	Behavioural problems emerged with emotional dysfunction and aggressivity. ADHD disorder
Spagnoli et al. (2022)	Case report • Age: 15 yo • Gender: F	Motor	-	• 24 mo: independent walking, with toe walking, frequent falls and disequilibrium • 15 yo: used a wheelchair to cover long distances. Diagnosed with pyramidal syndrome in her lower limbs, dyskinesia in her upper limbs and cerebellar signs
		Language	-	• 1 yo: first word (language delay and articulation and phonological impairment). Speech therapy
Taylor et al. (2022)	Case report • Age: 15 yo • Gender: F	Motor	-	• 5 mo: did not roll on either side • 9 mo: sat on her own • 10 mo: pulled up to stand • 5 yo: global developmental delay. Appropriate gross motor skills but delayed fine motor skills
		Language	-	• 5 yo: delayed speech
Yan et al. (2022)	*N* = 24 • Age range: 0.6–11 • Gender: F, 10; M, 14	IQ	Full-scale score unknown	• Intellectual disability/developmental disorder: 18 out of 24 • DQ ranging from 35 to 69
		Motor	Parents questionnaire	• Raising head: ranging from 1 to 6 mo • Sat independently: ranging from 8 to 18 mo • Standing independently: from 10 to 33 mo • Walked independently: from 13 to 37 mo • Gross motor delay: 24 out of 24 • Fine motor delay: 23 out of 24 • Gait abnormalities: 18 out of 22 • Myodystonia: 21 out of 24
		Language	Parents questionnaire	• Language delay: 5 out of 5 (age > 3) • No words: 4 out of 24 • Single words or phrases: 15 out of 24 • Short sentences: 1 out of 24
		Behaviour traits	Parents questionnaire	• Self-injury: 2 out of 24 • Impulsivity/irritability: 7 out of 24 • Anxiety: 8 out of 24 • Hyperactivity: 5 out of 24 • Sleep disturbances: 17 out of 24 (difficulties to fall asleep and night terror)
		Features of autism	Parents questionnaire	• Overly sensitive to touch/voice: 2 out of 24 • Repetitive behaviours: 8 out of 24 • Autism diagnosis: 3 out of 24
Zuluaga-Gómez et al. (2022)	Case report • Gender: M	IQ	-	• 3 mo: developmental delay
		Motor	-	• 3 mo: not able to sit upright, lacked good reflexes and exhibited axial and appendicular hypotonia
Chaves et al. (2023)	*N* = 1 • Age: 10 yo	IQ	-	• Mild NPMD • Intellectual disability
Ji et al. (2023)	*N* = 2 • Case 1: 8.25 yo/F • Case 2: 25 mo/M	IQ	-	Case 1: • 6 yo: started attending a special school. Parents refused to take the WISC Case 2: • Too young to take an intelligence test. Cognitive skills were delayed
		Motor	-	Case 1: • 8 mo: not able to raise her head or sit • 3 yo: walked independently (after rehabilitation training). She moved slowly with a scissor gait Case 2: • 3–4 mo: could hold his head • 6 mo: could turn over and hypotonia • 11 mo: crawl and could sit independently • 25 mo: slightly low muscle tone, hyperextension of the knee and bilateral knee tendon reflexes • 2 ½ yo: improvement of motor function. Could walk a short distance with support (broad-based gait) but had hypertonia of the legs and ankles
		Language	-	Case 1: • 4.5 yo: could not speak • 6.5 yo: She could tell a simple story and communicate with others but had repetitive language Case 2: • Delayed language development
		Behaviour traits	-	Case 1: • 4.5 yo: atypical behaviours, aggression towards others and emotional lability • 6.5 yo: improvement of the atypical behaviours
		Features of autism	-	Case 1: • 6.5 yo: stereotypic behaviours (patting objects)
Lee et al. (2023)	*N* = 2 • Case 1: 41 mo/F • Case 2: 12 mo/F	IQ	BSITD^b^	Case 1: • 17 mo: 4 mo of age in cognition • 41 mo: 8 mo of age in cognition Case 2: • 12 mo: 4 mo in cognition
		Motor	BSITD^b^; MASG^b^	Case 1: • 17 mo: 3 mo in fine motor skills and 2 mo in gross motor skills. Profound global developmental delay • 2 yo: could sit up • 41 mo: 8 mo in fine motor skills and 7 mo in gross motor skills. Severe developmental delay, dyskinetic movement and spasticity Case 2: • 12 mo: could roll over but could not sit alone. Spasticity in the lower limbs and was rated as Grade I + . Equivalent of 4 mo in fine motor skills and 4 mo in gross motor skills
		Language	BSITD^b^	Case 1: • 17 mo: 9 mo of age in expressive language and 9 mo of age in expressive language • 41 mo: 5 mo of age in receptive language and 6 mo in expressive language Case 2: • 12 mo: 4 mo in receptive language and 9 mo in expressive language
Moeinafshar et al. (2023)	*N* = 2 • Case 1: 7 yo/F • Case 2: 3 yo/F	IQ	-	Case 1: • No signs of intellectual disability Case 2: • Intellectual disability
		Motor	-	Case 1: • 13 mo: achieved assisted sitting • 3 yo 5 mo: achieved walking • 7 yo: axial hypotonia but no signs of spastic diplegia. Unable to run and jump, as well as severe ataxia. Occupational therapy helped improving Case 2: • 6 mo: able to hold head • 11 mo: able to sit independently • 7 yo: unable to walk, although occupational therapy helped improving. Axial hypotonia with no signs of spastic diplegia
		Language	-	Case 1: • 7 yo: speech impairment and manifestation of stuttering and inability to communicate verbally Case 2: • 7 yo: speech impairment with the inability to form sentences
		Features of autism	-	Case 1: • No signs of autistic feature Case 2: • Attention deficit and inability to communicate with peers
Onesimo et al. (2023)	*N* = 10 • Age range: 6–23 • Mean age: 10.8 yo • Gender: F, 2; M, 8	IQ	-	• Intellectual disability/developmental delay: 10 out of 10. Variable level of ID
		Motor	-	• Muscle tone abnormalities: 10 out of 10 • Hold head steady: mean age of 8.5 mo (range from 3 to 24 mo) • Trunk control: reached at mean age of 18 mo (range from 9 to 48 mo) • No crawling but sliding to another place: 1 out of 10 • Hypotonia of the trunk and hypertonia of the legs: 10 out of 10 • Walk independently: 7 out of 10 at the mean age of 3.5 y (range from 2 to 8) • Support of a walker: 3 out of 10 One case suffered a regression in walking ability due to the interruption of psychomotricity and physical rehabilitation during COVID-19
		Language	-	Language development was generally delayed • First word: mean age of 3 y (range from 1 to 4 y) • Build sentences and speak fluently: 5 out of 10 (at the time of the evaluation). The other half started to produce sentences when reaching the age of 5 y (range from 3 to 8 y) • Participation into a speech-language rehabilitation programme: 7 out of 10. One patient reported no significant improvement
		Features of autism	-	• Autism disorder: 1 out of 10
Sinibaldi et al. (2023)	*N* = 19 • Age range: 4–23 • Mean age: 10.3 yo • Gender: F, 8; M, 11	IQ	-	• Mild neurodevelopmental delay: 6 out of 19 • Moderate neurodevelopmental delay: 7 out of 19 • Severe neurodevelopmental delay: 6 out of 19 • Mild intellectual disability: 5 out of 19 • Moderate intellectual disability: 11 out of 19 • Severe intellectual disability: 3 out of 19
		Motor	-	Motor delay: 19 out of 19 • Truncal hypotonia: 4 out of 19 • Lower limb spasticity: 14 out of 19 • Dystonia: 4 out of 19 • Ataxic gait: 8 out of 19
		Language	-	• Speech delay: 19 out of 19
		Behaviour traits	-	• Aggressions/auto-aggressions: 7 out of 19 • Sleep disturbances: 6 out of 19 • ADHD: 7 out of 19
		Features of autism	-	• Autism: 7 out of 19
Nagy et al. (2024)	Case report • Age: 16 yo Gender: M	IQ	-	• Global developmental delay • Borderline IQ
		Motor	-	• Early childhood: axial hypotonia and increased tone in all 4 limbs • Currently: symptoms of complex movement disorder such as spasticity, slight ataxia, intermittent dystonia and stereotypes
		Behaviour traits	-	• Aggressiveness • Fits of anger
Sudnawa et al. (2024)	*N* = 32 • Age range: 1.8–22.7 • Mean age: 8.5 yo • Gender: F, 14; M, 18	IQ	DAS-II^b^; VABS^a^	Only 15 individuals completed the DAS-II • GCA scores: very low mean (*M* = 58.3) • Spatial score: very low mean (*M* = 47.5) • Verbal score: low mean (*M* = 73.6) • Nonverbal reasoning: low mean (*M* = 70.2) VABS mean score was 66.5 and 11/20 of the sample was classified as low adaptative functioning • Daily living skills: mean of 64
		Motor	GMFCS^b^; GMFM-66^b^; 10MWR^b^; 6MWT^b^; TUG^b^; RULM^b^; BBT^b^; 9HPT^b^; VABS^a^; MACS^b^	• GMFCS: 56% walked without an assistive device • GMFM-66: score of 56.6% % of sample that completed the tests: 10MWR (88.9%) 6MWT (72.2%), TUG (66.7%), RULM (71.9%), BBT (59.4%) and 9HTP (37.5%) • 10MWR: slower than normal (*M* = 6.5) and 56.3% had completion times more than 2 SD greater than normal • 6MWT: mean distance walked was 321.1 m • TUG: slower than normal (*M* = 9.6) • RULM: score of 27.6/37 • BBT: scores using the dominant hand were similar to published norms (*M* = 19) • 9HTP: slower than published norms (*M* = 51.8) • VABS (motor skills subdomain): mean of 66.1 • MACS: most individuals could handle objects without assistance
		Language	VABS^a^; CFCS^b^	• VABS (communication subdomain): mean of 68.2 • CFCS: 55% could communicate independently in most environments
		Behaviour traits	CBCL^a^; CSHQ^a^; QI-disability^b^	CBCL indicated that 65% of the sample has behavioural problems (17/32 completed the test) • Internalising problems: 47.1% (*M* = 61.5) • Externalising problems: 35.3% (*M* = 57.4) • Attention problems: (*M* = 62.8) CSHQ indicated that 66.7% has sleep problems (*M* = 44) (18/32 completed the test) QI-disability overall score was 81.7 • Negative emotions: mean of 74.5 • Positive emotions: mean of 90.3 • Physical health: mean of 86.1 • Social interaction: mean of 81.8 • Leisure and the outdoors: mean of 81.6 • Independence: mean of 75.6
		Features of autism	SCQ^a^; VABS^a^	• SCQ (15/32 completed the test): 33% of the sample had risk of social communication impairment • VABS (socialisation subdomain): mean of 66.9

The studies are described in chronological order

*ADHD*, attention-deficit/hyperactivity disorder; *ADOS-2*, Autism Diagnostic Observation Schedule; *AVZ-R*, Revised Scale for Autism and Related Disorders; *BBT*, Box and Blocks Test; *BSITD*, Bayley Scales of Infant and Toddler Development; *CBCL*, Child Behaviour Checklist; *CFCS*, Communication Function Classification System; *CSHQ*, Children Sleep Habits Questionnaire; *DAS-II*, Differential Ability Scales Second Edition; *DQ*, development quotient; *F*, female; *GCA*, General Conceptual Ability; *GMFCS*, Gross Motor Function Classification System; *GMFM*, Gross Motor Function Measure; *IQ*, intelligence quotient; *M*, male; *MACS*, Manual Ability Classification System; *MASG*, Modified Ashworth Scale Grade; *mo*, months; *N/A*, not applicable; *NPMD*, neuropsychomotor developmental delay; *QI-disability*, Quality of Life Inventory-Disability; *RULM*, Revised Upper Limb Module; *SCQ*, Social Communication Questionnaire; *SD*, standard deviation; *SED-R*, Dutch Scale for Emotional Development in People with Intellectual Disability; *TIQ*, total intellectual quotient; *TUG*, timed up and go; *VABS*, Vineland Adaptive Behaviour Scale; *WAIS*, Wechsler Adult Intelligence Scale; *WISC*, Wechsler Intelligence Scale for Children Fifth Edition; *yo*, years old; *6MWT*, 6-min walk test; *9HPT*, 9 Hole Peg Test; *10MWR*, 10-m walk run

^a^Parent-reported questionnaire

^b^In-person assessments by clinicians

### Risk of Bias

Risk of bias was evaluated for each eligible article using the adapted version of the modified Newcastle–Ottawa Scale (Bawor et al., 2014). No studies were excluded on the basis of risk of bias.

## Results

### General Overview

Out of the 42 articles chosen, 17 were case reports, 19 case series studies and 6 included in their research CTNNB1 syndrome patients along with others. Paediatric patients included from newborns to 17 years old, which tended to be sourced by the paediatric neurology department, and adults ranged from 18 to 51 years old and were drawn by the neurology department. A total of 15 articles provided longitudinal information on the evolution of the patients, although not always in the total recruited sample. The vast majority of studies included a sample below 20 participants, except for three articles (Kayumi et al., 2022; Sudnawa et al., 2024; Yan et al., 2022). The main limitation was the lack of instruments used to assess the variables considered, only nine referred the methodology used (Dashti et al., 2022; Jin et al., 2020; Lee et al., 2023; Lee et al., 2022; Pipo-Deveza et al., 2018; Sudnawa et al., 2024; Takezawa et al., 2018; Verhoeven et al., 2020; Wang et al., 2019). No article had a control group to compare results, and only Sudnawa et al. (2024) carried out a statistical analysis to compare mean differences of cognitive and adaptative functioning domains. Overall, even having found 42 articles in the literature, risk of bias was high in most studies calling into question the veracity of the results found (Table 3). The following results are specifically based on those articles that have assessed the chosen variables with a standardised methodology, which have been marked in Table 3.

**Table 3:** Risk of bias assessment for reviewed studies

	Method for selecting sample	Methods to control confounding		Statistical methods		Methods for measuring outcomes	
		Sample size	Identification of confounders	Appropriate analyses	Missing data	Outcomes measure	Objective assessment
Dubruc et al. (2014)	↑	↑	↑	↑	↓	↑	↑
Tucci et al. (2014)	↑	↑	↑	↑	↓	↑	↑
Kuechler et al. (2015)	↑	↔	↑	↑	↓	↑	↑
Dixon et al. (2016)	↑	↑	↑	↑	↓	↑	↑
Prasad et al. (2016)	↑	↑	↑	↑	↓	↑	↑
Retterer et al. (2016)	↑	↑	↑	↑	↓	↑	↑
Thevenon et al. (2016)	↑	↑	↑	↑	↓	↑	↑
Winczewska-Wiktor et al. (2016)	↑	↑	↑	↑	↓	↑	↑
Kharbanda et al. (2017)	↑	↔	↑	↑	↓	↑	↑
Krupp et al. (2017)	↑	↑	↑	↑	↓	↑	↑
Li et al. (2017)	↑	↑	↑	↑	↓	↑	↑
Panagiotou et al. (2017)	↑	↑	↑	↑	↓	↑	↑
Cordeiro et al. (2018)	↑	↑	↑	↑	↓	↑	↑
Percy et al. (2018)	↑	↑	↑	↑	↓	↑	↑
Pipo-Deveza et al. (2018)*	↑	↑	↑	↑	↓	↔	↔
Takezawa et al. (2018)*	↑	↑	↑	↑	↓	↔	↔
Sun et al. (2019)	↑	↑	↑	↑	↓	↑	↑
Wang et al. (2019)*	↑	↑	↑	↑	↓	↔	↔
Coussa et al. (2020)	↑	↑	↑	↑	↓	↑	↑
Jin et al. (2020)*	↑	↑	↑	↑	↓	↔	↔
Ke and Chen (2020)	↑	↑	↑	↑	↓	↑	↑
Rossetti et al. (2020)	↑	↑	↑	↑	↓	↑	↑
Verhoeven et al. (2020)*	↑	↑	↑	↑	↓	↔	↔
Yan et al. (2020)	↑	↑	↑	↑	↓	↑	↑
Bulot et al. (2022)*	↑	↔	↑	↑	↓	↑	↔
Dashti et al. (2022)*	↑	↑	↑	↑	↓	↔	↔
Ho et al., (2022a, 2022b)	↑	↑	↑	↑	↓	↑	↑
Kayumi et al. (2022)	↑	↓	↑	↑	↓	↑	↑
Lee et al. (2022)	↑	↔	↑	↑	↓	↑	↑
Paparella et al. (2022)	↑	↑	↑	↑	↓	↑	↑
Spagnoli et al. (2022)	↑	↑	↑	↑	↓	↑	↑
Taylor et al. (2022)	↑	↑	↑	↑	↓	↑	↑
Yan et al. (2022)*	↑	↔	↑	↑	↓	↑	↔
Zuluaga-Gómez et al. (2022)	↑	↑	↑	↑	↓	↑	↑
Chaves et al. (2023)	↑	↑	↑	↑	↓	↑	↑
Ji et al. (2023)	↑	↑	↑	↑	↓	↑	↑
Lee et al. (2023)*	↑	↑	↑	↑	↓	↔	↔
Moeinafshar et al. (2023)	↑	↑	↑	↑	↓	↑	↑
Onesimo et al. (2023)	↑	↔	↑	↑	↓	↑	↑
Sinibaldi et al. (2023)	↑	↔	↑	↑	↓	↑	↑
Nagy et al. (2024)	↑	↑	↑	↑	↓	↑	↑
Sudnawa et al. (2024)*	↑	↔	↑	↔	↓	↓	↓

Quality indicators have been selected following the adapted version of modified Newcastle–Ottawa Scale (Bawor et al., 2014)

↑, high risk of bias; ↔ , moderate risk of bias; ↓, low risk of bias

^*^Studies with standardised methodology

### Intellectual Quotient

IQ refers to measures that estimate global cognition. Six articles administered general cognition batteries in patients diagnosed with CTNNB1 syndrome.

Tests selected for paediatric use were the Wechsler Intelligence Scale for Children—Fifth Edition (WISC-V) (Dashti et al., 2022), the Bayley Scales of Infant and Toddler Development (BSITD) (Lee et al., 2023), the Differential Ability Scales Second Edition (DAS-II), the Vineland Adaptative Scale (VABS) (Sudnawa et al., 2024) and an ad hoc online questionnaire (Bulot et al., 2022). Although the VABS is a measure of adaptive functioning, it is included in the IQ section as it provides valuable information about the performance in many aspects of everyday life.

Based on the four articles that have been mentioned, Dashti et al. (2022) demonstrated a non-progressive low average score in the WISC-V, with a total score of 80–89. Lee et al. (2023) described longitudinal information about cognition, assessing one case at 17 and 41 months of age, who had an age equivalent cognition of a 4 and 8 months old, respectively. Moreover, the authors described another case assessed at 12 months of age scored and age equivalent cognition to a 4 months old. Additionally, Bulot et al. (2022) determined varying degrees of intellectual impairment through an online questionnaire; 8 out of 12 had ID, 4 out of 8 had severe learning difficulties and the remaining 4 had some learning difficulties but most could read and do some simple maths. Only Sudnawa et al. (2024) provided domain-specific information of the DAS-II. 73.3% of the sample obtained *very low* scores on the general concept ability, whereas the remaining 26.7% were classified as *low*. Regarding the verbal domain, 33.3% indicated *very low* functioning and 20% *low* abilities, although 20% of the sample scored *below average* and 26.7% obtained *average* scores. The following domain was the nonverbal reasoning, in which 46.6% obtained *very low* scores and the 40% scores were classified as *low*. However, 6.7% indicated *below average* functioning and the remaining 6.7% had scores on the *average* range. Spatial domain was the most affected domain, where 93.3% of the sample obtained *very low* punctuations and the remaining 6.7% were classified as *low* scores. In fact, this domain was significantly lower compared to the verbal and nonverbal domains. Moreover, Sudnawa and colleagues (2024) described a mean score of 64 on the daily living skills of the VABS, indicating significant skill deficit when compared with similarly aged peers. All VABS subdomains were equally affected, with no significant differences across subdomains.

On the other hand, tests selected for adult participants were the VABS (Verhoeven et al., 2020) and the Wechsler Adult Intelligence Scale (WAIS) (Wang et al., 2019). Both studies reported either ID or developmental delay; Wang and colleagues (2019) reported severe ID with IQ scores of 28 and 40, whereas Verhoeven et al. (2020) described a 37-year-old who scored in the daily activities’ domain of the VABS a 44 months developmental age score.

The remaining 36 studies did not refer what instrument was used to assess IQ but provided information related to this variable. Nonetheless, all the articles reported ID or developmental delay with different severity degrees.

### Motor

Motor assessment refers to measures that study gross and fine motor development. Eight articles include the instruments used to assess motor development.

Specifically, three articles included longitudinal information about the progression of motor development. Pipo-Deveza et al. (2018) described a 4-year-old with delayed gross motor skills who was administered a treatment of L-dopa during 18 months. Overall, the participant had a significant reduction of hypertonia on the Tardieu scale and improved the scores obtained at the Gross Motor Function Measure (GMFM88), standing (51 to 69%) and walking/running (17 to 44%). Scores on the 6-min walk test (6MWT) also increased from 4.5 to 286 m. The treatment also accelerated the acquisition of fine motor skills. On the other hand, Verhoeven et al. (2020) reported the case of a 32-year-old who experienced a regression at a motor level, being wheelchair dependant at the current age. The participant obtained a punctuation in accordance with 34 months old on the motor skills domain of the VABS. Another study used the BSITD to assess two CTNNB1 syndrome patients. The first patient was assessed at 17 and 41 months and obtained and age equivalent of 3 and 8 months in fine motor skills and 2 and 7 months in gross motor skills, respectively. The following patient had spasticity in the lower limbs and was rated as Grade I + in the Modified Ashworth Scale. On the BSITD, her gross motor skills corresponded to an equivalent of a 4-month-old (Lee et al., 2023).

The other five articles used a cross-sectional design to assess motor development. Jin et al. (2020) used the Gross Motor Function Classification System (GMFCS) to assess two patients, who scored a level 2 and 3. Both participants presented spasticity, although the first one also had dystonia, gait abnormality and used an ankle–foot orthosis (AFO). The case report assessed by Takezawa et al. (2018) also used the GMFCS and obtained a score of 4, indicating limited self-mobility, together with the Manual Ability Classification System (MACS), whose punctuation was 3, consequently having difficulties in handling objects.

Another study used an ad hoc parents’ questionnaire to study the motor milestones. Overall, the whole sample presented gross motor delay, and almost everyone had fine motor delay. Moreover, the age of achievement of the most significant motor milestones was delayed in most patients (Yan et al., 2022). Bulot et al. (2022) described the whole sample with motor delay and gait disorders. However, almost all the patients were able to sit freely at the mean age of 17 months old and started walking at an age range from 24 months to 14 years old. Sudnawa et al. (2024) used an extensive assessment protocol for motor development; however, not all of their cohort was assessed due to difficulties the participants experienced with following instructions. The mean score of the GMFM66 was 56.6%, and the motor skills subdomain of the VABS scored a mean of 66.1, so both tests indicated general motor impairment. Ability to walk was assessed by the GMFCS, 6MWT, the 10-m walk run (10MWR) and the timed up and go (TUG). In this case, 56% of the sample walked without an assistive device, whereas the distance walked on the 6MWT was 321.1 m and scores on the remaining instruments indicated a walking pattern slower than normal. Upper limb function was assessed with the Revised Upper Limb Module (RULM), the Box and Blocks Test (BBT), the 9 Hole Peg Test (9HPT) and the MACS. The average score on the RULM was 27.6/37, and the BBT scores using the dominant hand were similar to published norms, although the 9HPT performance was slower than average. The MACS showed that most individuals could handle objects without assistance.

The remaining 35 articles did not mention the instrument used to assess motor development; the vast majority described the symptomatology based on clinical manifestations. All articles described motor delay or impairment with significant variety levels of affection, from patients unable to walk, some in need of an aid and the remaining could walk but showed cerebellar signs. The most common signs reported on the literature were hypotonia and spasticity, mainly affecting the lower extremities bilaterally, and ataxic gait. Nevertheless, the vast majority of patients achieved the most significant motor milestones later than expected and with several difficulties; hence, a slow but progressive improvement was seen over time. This was not the case for the two adult patients with longitudinal information on the motor domain, as they reported motor regression (Tucci et al., 2014; Verhoeven et al., 2020).

### Language

Language refers to the ability to understand (receptive language) and the ability to speak (expressive language). Only six articles mentioned the method used to assess this domain.

The communication domain of the VABS, which includes expressive, receptive and written communication, was used by Verhoeven et al. (2020) on a 32-year-old case report and Sudnawa et al. (2024) on a cohort of 32 participants. The first authors described a communication level in accordance with a 34-month-old, whereas the second researchers obtained a raw mean score of 68.2, indicating a general skill deficit. The BSITD was used by Lee et al. (2023) to assess two patients. The first one had an equivalent of 9 months in receptive and expressive language at the age of 12 months, although results worsened at the age of 41 months, scoring an age equivalent of 5 months in receptive language and 6 months in expressive language. The second case was assessed at 12 months old and scored an age equivalent of 4 months in receptive language and 9 months in expressive language. Sudnawa and colleagues (2024) also determined that 55% of the sample could communicate independently in most environments by assessing the Communication Function Classification System (CFCS). In the same line, Takezawa et al. (2018) described a 7-year-old with level 3 on the CFCS, so that he/she could be and effective sender and receiver with known interlocutors during a conversation. On the other hand, Yan and colleagues (2022) used an ad hoc questionnaire to assess the language domain. The severity of the impairment varied among the sample; 4 out of 24 had no words, 15 out of 24 could say single words or phrases and only 1 individual could speak in short sentences. Bulot et al. (2022) also used the same methodology and mentioned that, although all patients had speech delay, most of them acquired progressively expressive language milestones. In any case, articulation difficulties were present in most patients. Regarding receptive language, most of the sample reported a good level, even some patients understood complex orders.

The vast majority of the articles reviewed did not mention what instrument was used to assess language matters, although 36 of them provided information related to this topic. All studies described that language development was generally delayed, even though there were different degrees of impairment, from nonverbal to normal speech development with some articulation impairments such as dyspraxia. Therefore, individuals acquired language milestones, specifically expressive abilities, later than expected. Some of those individuals who had minimal expressive language communicated with signs or pictograms (Panagiotou et al., 2017; Tucci et al., 2014). Other studies reported improvements over time thanks to speech therapy (Dashti et al., 2022; Spagnoli et al., 2022; Verhoeven et al., 2020), although worsening of expressive and receptive abilities was noted in other studies (Percy et al., 2018; Pipo-Deveza et al., 2018). Some research pointed out that individuals had better comprehension than expressive skills (Dubruc et al., 2014; Verhoeven et al., 2020; Winczewska-Wiktor et al., 2016).

### Behavioural Traits

Behavioural problems refer to those behaviours and emotional disturbances considered characteristics of a behavioural disorder. Only three articles referred to the methodology used to assess behavioural traits.

Sudnawa et al. (2024) used the Child Behaviour Checklist (CBCL), the Children Sleep Habits Questionnaire (CSHQ) and the Quality of Life Inventory-Disability (QI-disability). The first instrument indicated that 65% of the sample had behavioural problems and, more specifically, internalising problems were more prevalent than externalising problems, 47.1% and 35.3%, respectively. Furthermore, 12 individuals had sleep problems based on the CSHQ, the most common problem being short sleep duration and parasomnia. In third place, the mean overall score of quality of life was 81.7 on the QI-disability, and the negative emotions subdomain (mean of 74.5) was significantly lower than the positive emotions subdomain (mean of 90.3). Both Bulot et al. (2022) and Yan et al. (2022) used an ad hoc online questionnaire to assess this matter. The first authors described temper tantrum as the most prevalent symptomatology (9 out of 12), followed by aggressions or auto-aggressions (6 out of 12), good social interactions and being a quiet child (4 out of 12) and, at the last place, attention-deficit/hyperactivity disorder (ADHD) in two patients. In contrast, Yan et al. (2022) found sleep disturbances were the most prevalent symptom (17 out of 24), specifically difficulties to fall asleep and night terrors, in second place anxiety (8 out of 24), impulsivity/irritability (7 out of 24), followed by hyperactivity (5 out of 24) and, finally, self-injury (2 out of 24).

The remaining 39 articles did not refer what instrument or methodology was used to assess behaviour traits. No further details other than the already reported were given, although Verhoeven et al. (2020) stressed that disruptive behaviours worsened when the usual routine changed. In addition, daily guidance resulted in significant reduction of challenging behaviours.

### Features of Autism

Features of autism refer to those behaviours and symptomatology that are associated with ASD. Four articles described the methodology used to assess this matter, although only two used a standardised test.

On the one hand, Verhoeven et al. (2020) included a psychological protocol based on the following instruments: the VABS, Dutch Scale for Emotional Development in People with Intellectual Disability (SED-R), the Revised Scale for Autism and Related Disorders (AVZ-R) and the Autism Diagnostic Observation Scale (ADOS-2). The case report was assessed at 32 years of age and scored a developmental age equivalent of 18 months (SED-R) and also at the socialisation domain of the VABS. Both instruments, the AVZ-R and the ADOS-2, corroborated the presence of symptomatology compatible with an ASD diagnosis. On the other hand, Sudnawa et al. (2024) administered the socialisation subdomain of the VABS and the Social Communication Scale (SCQ). The VABS socialisation mean score was 66.9, indicating impairment in interpersonal relationships, play and leisure activities and coping skills in social situations, whereas the SCQ showed that 33% of the sample had risk of social communication impairment. The other studies designed an ad hoc protocol to ask for this matter. Bulot and colleagues (2022) determined that the most prevalent symptoms were repetitive behaviours (8 out of 12), interests in specific things (6 out of 12), little eye contact (4 out of 12) and two had an ASD diagnosis. Similarly, Yan et al. (2022) described repetitive behaviours (8 out of 24), overly sensitive to touch or voice (2 out of 24) and three were previously diagnosed with ASD.

The other 38 articles did not specify the instrument used to assess features of autism. However, some of them provided information related to this topic. In fact, some of the patients reported had been previously diagnosed with ASD (Onesimo et al., 2023; Rossetti et al., 2021; Sinibaldi et al., 2023; Sun et al., 2019). The manifestations reported were on the same page as the ones previously described, such as avoidance of eye contact (Kuechler et al., 2015; Percy et al., 2018; Winczewska-Wiktor et al., 2016), stereotypic movements (Ji et al., 2023; Kharbanda et al., 2017; Kuechler et al., 2015; Lee et al., 2022; Rossetti et al., 2021; Winczewska-Wiktor et al., 2016) and signs of frustration (Winczewska-Wiktor et al., 2016). In contrast, two studies specifically reported no signs of features of autism (Moeinafshar et al., 2023; Takezawa et al., 2018).

## Discussion

Although CTNNB1 syndrome has always been described as a neurodevelopmental disorder which consists of cognitive and behavioural impairments, the focus on previous literature has typically been genetic matters and description of symptoms. Only a few articles have used standardised methods to evaluate some of the main clinical manifestations of CTNNB1 syndrome, which include ID, motor impairments, language delay, behavioural problems and features of autism. The instruments used for assessment can be classified according to whether they were applied to the parents or directly to the individuals with CTNNB1 syndrome. Some authors state the inability to assess neuropsychological instruments or motor test that require the participation of the individual due to a very low cognitive functioning (Sudnawa et al., 2024). Moreover, the eligible studies have numerous limitations. Firstly, sample size was small in the vast majority of studies and most patients were paediatric individuals; hence, information related to adults is scarce, which has also been described in other reviews (Ho et al., 2022a, 2022b). This fact could be explained due to the recent discovery of the syndrome, just over 10 years ago by de Ligt et al. (2012), and that the only reliable way to establish a diagnosis is from a whole-exome or genome sequencing analysis (Dashti et al., 2022), which is increasingly being utilised in the investigation of genes implicated in ID, speech and language disorders, ASD and cerebral palsy (Basu et al., 2024; Gonzalez-Mantilla et al., 2023; Kharbanda et al., 2017; Srivastava et al., 2019). Kayumi and colleagues (2022) determined that an early application of exome sequencing could have prevented unnecessary testing and provided a quicker diagnosis. Several patients reported on the literature had been previously diagnosed with cerebral palsy or autism, due to the analogies in symptomatology and lack of genetic testing (Jin et al., 2020; Lee et al., 2023; Sun et al., 2019; Verhoeven et al., 2020). It is therefore logical to assume that there are potentially adults with CTNNB1 syndrome who have not had access to genetic testing. Alongside with the methodological heterogeneity, there is also diversity regarding the clinical picture of the CTNNB1 syndrome patients reported so far. Only Sudnawa et al. (2024) designed an extensive neuropsychological and clinical protocol tailored to the needs of CTNNB1 syndrome individuals and carried out statistical analysis. However, no study included a control group. To our knowledge, no critical analysis about these specific matters has previously been performed, even though other reviews have focused on addressing the general aspects surrounding CTNNB1 syndrome (Ho et al., 2022a, 2022b; Miroševič et al., 2022; Zhuang et al., 2023).

Considering each specific domain, all articles described general ID among participants with varying degrees of impairment, both paediatric and adult patients. This finding goes in line with other investigations, establishing ID as one of the main clinical manifestations of the syndrome (Ho et al., 2022a, 2022b; Miroševič et al., 2022; Zhuang et al., 2023). Specifically, those that used standardised instruments also corroborated this fact. Some longitudinal studies showed progressive but low improvements, specifically with paediatric participants (Lee et al., 2023). One of the main limitations regarding this domain is that studies only provided general or mean raw scores of the test assessed, making it difficult to interpret the results (Dashti et al., 2022; Sudnawa et al., 2024; Wang et al., 2019). Moreover, certain standardised batteries, such as the WISC-V or the WAIS, do not take into consideration some of the clinical specificities of the CTNNB1 syndrome individuals, making them unsuitable for assessment (Dashti et al., 2022; Wang et al., 2019). Sudnawa et al. (2024) reported scores for specific domains and concluded that the spatial variable was the most affected domain. In fact, scores were significantly lower compared to nonverbal and verbal domain, in which some obtained average scores. Nevertheless, CTNNB1 syndrome individuals have significant visual impairments which could interfere when performing visual tasks, so that verbal and nonverbal domains are more likely to give accurate information. To date, this is the only study that has traced in a preliminary way the cognitive profile associated to the CTNNB1 syndrome.

Secondly, motor development was the variable studied for which the most information has been obtained, mostly based on clinical descriptions. First of all, age achievement of the most significant milestones was delayed in most patients (Bulot et al., 2022; Yan et al., 2022). In particular, Ho et al., (2022a, 2022b) described truncal hypotonia as the first sign of motor impairment, which goes in line with the fact that CTNNB1 syndrome newborns take more time to hold their heads or sit with support due to the decreased muscle tone. The same authors also suggest that this symptom could be the first alert sign to prompt for medical attention. In the second place, different degrees of impairment regarding motor functioning were found among patients (Jin et al., 2020; Takezawa et al., 2018). This goes in line with the presence of muscle tone abnormalities such as dystonia, extremities hypertonia, muscle weakness or gait abnormalities (Ho et al., 2022a, 2022b; Miroševič et al., 2022). To our knowledge, no studies have analysed how early acquisition of motor milestones can predict the severity of motor symptomatology, although in analogous diseases, such as cerebral palsy, it is well known that an early acquisition of the gross motor milestones sitting predicts walking ability since antigravity muscles for the trunk or postural control are fundamental for the upright position development (Keeratisitoj et al., 2018). Another relevant finding was that gross motor impairments were more significant than fine motor skills (Sudnawa et al., 2024; Takezawa et al., 2018). Contradictory results have been found among longitudinal studies, while patients described improvement (Lee et al., 2023) and gross motor scales positively correlated with age, suggesting continued gain of motor skills (Sudnawa et al., 2024); the only two adult patients reported presented motor regression (Tucci et al., 2014; Verhoeven et al., 2020). Future research should focus on studying the progression of motor symptomatology, especially in adult patients, bearing in mind that preliminary studies may indicate a worsening over time. Finally, Pipo-Deveza et al. (2018) described a possible therapeutic application with encouraging results not only on the motor side but also in cognition and behaviour.

The language variable has been one of the least reported domains. In fact, only Sudnawa et al. (2024) have directly assessed the CTNNB1 syndrome patients, while the other studies have used standardised instruments to assess parents, so the information is not as precise and specific. The vast majority had speech delay with impairments related to articulation problems such as dyspraxia. Acquired language milestones were later than expected, specifically for expressive abilities, as it was corroborated by the communication domain of the VABS and the BSTID (Lee et al., 2023; Sudnawa et al., 2024; Verhoeven et al., 2020). CTNNB1 syndrome individuals exhibit varying degrees of expressive language, from nonverbal to verbal with articulation impairments (Ho et al., 2022a, 2022b; Miroševič et al., 2022), in line with the other manifestations of the syndrome. Instruments showed that individuals could communicate independently in most environments, even though communication was more effective and fluent with close people (Sudnawa et al., 2024; Takezawa et al., 2018). In fact, Sudnawa et al. (2024) found the verbal domain of the DAS-II to be the most preserved domain compared to nonverbal and spatial functioning. Moreover, some nonverbal patients used sign language or pictograms to communicate (Panagiotou et al. 2017; Tucci et al., 2014). On the other hand, receptive language was, in most patients, preserved and even some patients understood complex orders. Other studies pointed out that receptive language was significantly better than expressive (Ho et al., 2022a, 2022b; Miroševič et al., 2022). 

Behavioural domain is also one of the least studied variables, with only three articles referring to the methodology used. Therefore, vague information was obtained, making it difficult to describe the behavioural profile associated with the CTNNB1 syndrome. Still, Sudnawa and colleagues (2024) were the only ones to assess behavioural problems on the basis of an assessment protocol. The main findings were that internalising problems were more frequent than externalising problems, contrary to what descriptive studies reported (Ho et al., 2022a, 2022b; Miroševič et al., 2022). As the authors point out, diagnosis of mood disorders in individuals with ID can usually be misdiagnosed as they have less ability to communicate and express feelings. Also, caregiver questionnaires can sometimes be influenced by the parents’ perception. Another relevant behavioural symptom reported was sleep problems (Sudnawa et al., 2024; Yan et al., 2022), which correlated significantly with total score problems, indicating more overall behavioural problems when having sleep difficulties. This is a very significant finding that considers the importance of dealing with sleep problems, which had previously been considered a secondary symptom. Future research should also focus on the impact of sleep problems, not only on behaviour but on general development aspects as well. Separately, quality of life was assessed to a sample of CTNNB1 syndrome individuals and indicated that overall score was higher compared to other neurodevelopmental disorders (Sudnawa et al., 2024), stressing the importance of daily guidance and building good emotional support to reduce challenging behaviours and improve day-to-day life independence (Sudnawa et al., 2024).

Features of autism have also been reported. Only Verhoeven et al. (2020) and Sudnawa et al. (2024) used a specific protocol to assess this matter. Contradictory results were found among screening tools for autistic signs, which highlights the heterogeneity of the patients assessed. Studies that used age equivalent test all suggest a significant delayed age equivalent for the socialisation domain (Verhoeven et al., 2020). Future research should use instruments that allow for a description of the features of autism of CTNNB1 syndrome. Nevertheless, other studies determined that some of the most prevalent symptoms included repetitive behaviours, interest in specific things, little eye contact and overly sensitive to touch or voice (Bulot et al., 2022; Yan et al., 2022). Some patients had an ASD diagnosis which highlights the analogy of ASD with CTNNB1 syndrome and the importance of genetic testing in neurodevelopmental disorders (Srivastava et al., 2019). Due to insufficient information reported, it is very difficult to obtain conclusive results. However, it is assumed that the presence of ASD symptomatology would vary from case to case, although it is reported as a common clinical manifestation (Ho et al., 2022a, 2022b; Miroševič et al., 2022; Zhuang et al., 2023). In fact, *CTNNB1* has been identified as an ASD-associated gene (Zhuang et al., 2023).

Having reviewed the eligible studies, many limitations need to be mentioned. First of all, sample size was small in the vast majority of articles or presented single-case reports. Likewise, only one article included statistical analysis and no research included a comparative control group. In addition, adult patients were very few, which implies that there are many unknowns about the progression of the syndrome later in life probably due to misdiagnosis. The main limitation found was the lack of a standardised methodology to assess cognitive and behavioural domains, which calls into question the veracity of the results and the replicability of the studies. In addition to this, some of the studies that described the methodology used had selected instruments that are not suitable for CTNNB1 syndrome individuals. Specifically, they did not consider the possible motor, visual or verbal difficulties associated to the syndrome. Most studies did not provide specific information, especially on cognitive aspects, which would be the most relevant data to characterise the cognitive profile. A list of recommended elements and methods for the assessment of CTNNB1 syndrome patients that could be applied to future studies and clinical practice is summarised in Table 4.

**Table 4:** Recommendations for assessing CTNNB1 syndrome patients for clinical and research purposes

General	• The assessment should be divided into short parts in an attempt to maximise attention • Longitudinal assessments would provide valuable information on the progressions of the disease • If possible, the examinee should be familiarised with the examiner and the context of the assessment so that they feel comfortable • Results could be bias due to visual defects associated to the CTNNB1 syndrome
Cognition	• Parent-reported questionnaires should be an addition to in-person neuropsychological assessments • Neuropsychological assessments should include nonverbal tasks • Instruments should not imply a high motor performance • Parent-reported questionnaires should focus on evaluating daily live functioning • The tests protocols should be adapted to the developmental age of the patient
Language	• Separately assess expressive language ability, as some patients may be able to communicate • Assessment of intelligibility, articulation, oral motor structure functioning and phonology would provide overall speech accuracy. The assessment should be carried out by a qualified speech therapist • Vocabulary screening tests would be an easy and effective option to assess expressive language ability • Parent-reported questionnaires should focus on assessing overall communication ability • Achievement of expressive language milestone would be relevant information
Motor	• Assessments of gross and fine motor skills should be carried out by qualified physiotherapist • Gross motor should focus on assessing the level of gross motor skills achieved, upper limb function and ambulatory function • Related to fine motor skills, it would be important to assess grip strength and quality, eye-hand coordination, use of pincer grasp and quality in object handling • Parent-reported questionnaires should focus on assessing manual skills on day-to-day activities • Achievement of gross and fine motor milestones would be relevant
Behaviour	• Parent-reported questionnaires should assess both internalising and externalising problems • Sleep problems should also be assessed using screening questionnaires
Autism features	• Instruments that focus on detection symptomatology compatible with ASD would be useful, instead of screening tests

## Conclusions

CTNNB1 syndrome is characterised by a pervasive neurodevelopmental delay that affects many aspects of a person’s life. Currently, the vast majority of studies with CTNNB1 syndrome patients have a medical vision, leaving behind aspects such as cognition or behaviours, which are only mentioned as additional symptoms of the clinical examination. For this reason, the information available on the topic is imprecise, which limits an extensive description of the syndrome and generalizable to all patients. In recent years, the literature related to CTNNB1 syndrome has increased, along with the improvements on the methodology followed. In fact, the first study to count with a specific assessment protocol has been recently published by Sudnawa et al. (2024). Future research should consider this research for the design of an assessment protocol. In turn, it is also essential that forthcoming investigations analyse the interaction of neurodevelopmental variables to target therapeutic approaches and include analogous comparative control groups to establish differences between disorders.

## Data Availability

This review is registered at PROSPERO under the following identification number: CRD42024508864.
